# 25487 Timing and strength of hand grasp that are affected by abnormal coupling between arm muscles following stroke: A pilot study

**DOI:** 10.1017/cts.2021.495

**Published:** 2021-03-30

**Authors:** Dongwon Kim, Kyung Koh, Raziyeh Baghi, Li-Chuan Lo, Chunyang Zhang, Giovanni Oppizzi, Dali Xu, Li-Qun Zhang

**Affiliations:** University of Maryland, Baltimore

## Abstract

ABSTRACT IMPACT: Impaired neuromuscular control could lead to the failure in activation and deactivation of the target muscles in a timely manner, with the concurrence of activities of non-targeted muscles. OBJECTIVES/GOALS: Stroke leads to impaired capacity to manipulate objects with the hand in terms of timing and strength of grasp. The influence of abnormal coupling across more proximal arm muscles post stroke on the failure in normal functioning of finger flexor muscle activity is of interest to investigate. METHODS/STUDY POPULATION: We have recruited 12 participants with stroke hemiparesis in the sub-acute or chronic stage. Motor impairment of the arm was assessed using electromyography (EMG) and the Fugl-Meyer Upper Extremity (FMUE) assessment. Participants were requested to flex and relax the metacarpophalangeal (MCP) joints against motorized resistance in response to audible tones to determine timing and strength during flexion. They were asked to flex maximally, as quickly as possible, in response to the first of a pair of tones, and relax as quickly as possible after the second tone. Delays in initiation and termination were evaluated using EMG responses versus a predefined threshold. RESULTS/ANTICIPATED RESULTS: We anticipate greater delays in grasp initiation as well as in grasp termination in participants with a greater extent of abnormal coupling across more proximal muscles of the upper extremity in comparison to participants with a less extent, using the results of the FMUE assessment. Also it is expected that participants with a greater extent of the flexion synergy produce a less extent of force generation. The EMG results will show that activities of more proximal arm muscles precede the initiation of MCP flexion and their activity termination precedes that of MCP flexion, significantly more in participants with a greater extent of the flexion synergy. DISCUSSION/SIGNIFICANCE OF FINDINGS: The flexion synergy over the arm following stroke affects the timing and strength of hand grasp. Impaired neuromuscular control could lead to the failure in activation and deactivation of the target muscles in a timely manner, with the concurrence of activities of non-targeted muscles.

